# The complete mitochondrial genome of the Chinese noodlefish *Leucosoma chinensis* and phylogenetic analysis of Salangidae (Osteichthyes: Osmeriformes)

**DOI:** 10.1080/23802359.2020.1860720

**Published:** 2021-01-27

**Authors:** Fei Xiong, Dongdong Zhai, Jixin Yu, Yuanyuan Chen, Ying Wang, Hongyan Liu

**Affiliations:** a School of Life Sciences, Hubei Engineering Research Center for Protection and Utilization of Special Biological Resources in the Hanjiang River Basin, Jianghan University, Wuhan, China; b Hubei Key Laboratory of Environmental and Health Effects of Persistent Toxic Substances, Jianghan University, Wuhan, China

**Keywords:** *Leucosoma chinensis*, *Salanx chinensis*, mitochondrial genome, Salangidae, phylogenetic tree

## Abstract

In the present study, we obtained the first complete mitochondrial genome sequence of *Leucosoma* (*Salanx*) *chinensis*. It was 16,595 bp in length and consisted of 13 protein-coding genes, 22 transfer RNA genes, 2 ribosomal RNA genes, and a non-coding control region. The nucleotide composition was A (23.1%), T (25.2%), G (20.0%) and C (31.8%), and the A + T content (48.3%) was a little lower than G + C content (51.80%). Phylogenetic analysis of 10 species of Salangidae identified three major clades. These results may facilitate the future genetic research of *L. chinensis* and Salangidae.

The Chinese noodlefish *Leucosoma chinensis* (Salangidae, Osmeriformes, Osteichthyes) is endemic to east and south Chinese seas (Zheng 1989). It is sometimes classified in the genus *Salanx* (e.g. Froese and Pauly 2019). It has a slender body, a very flat head, a long snout, and a small fleshy protrusion on the front of the lower jaw. Each fin is small, the dorsal fin is located above the anal fin, and there is a pair of small transparent adipose fins between the dorsal and caudal fins. Its body is soft and transparent without scales. It usually lives in coastal waters, and reproduces in brackish or fresh water during the reproductive period. It mainly feeds on zooplankton. Members of this species are small, and the meat is considered delicious and nutritious (Su and Wang 1985; Zheng 1989; Huang et al. 2012). In the present study, we obtained the first complete mitochondrial genome sequence of *L. chinensis*, which may facilitate future genetic research on this species.

A sample of *L. chinensis* was collected from the Wuzhou (N23°28′23″, E111°22′16″) section of the Xijiang River in 2019. The fish was placed in 95% alcohol and reposited in a refrigerator at −20 °C in the specimen room of the School of Life Sciences, Jianghan University (Sample code: *L. chinensis* 20190712001). Total genomic DNA from the dorsal muscle was extracted using the Foregene Animal Tissue DNA Kit. The complete mitochondrial genome was obtained from Illumina high-throughput sequencing and *de novo* assembled using NOVOPlasty ver 2.6 (Dierckxsens et al. 2017). The tRNA genes were predicted using the program ARWEN (Laslett and Canback 2008), rRNA and protein-coding genes were created using web server DOGMA (Wyman et al. 2004).

The complete mitochondrial genome of *L. chinensis* was 16,595 bp in length (GenBank accession number: MW131880). The nucleotide composition was A (23.1%), T (25.2%), G (20.0%) and C (31.8%), and the A + T content (48.3%) was a little lower than G + C content (51.80%). The complete mitochondrial genome consisted of 13 protein-coding genes, 22 transfer RNA genes, 2 ribosomal RNA genes, and a non-coding control region. *ND6* gene and 8 tRNA genes (*tRNA^Pro^*, *tRNA^Glu^*, *tRNA^Ser^*, *tRNA^Tyr^*, *tRNA^Cys^*, *tRNA^Asn^*, *tRNA^Ala^* and *tRNA^Gln^*) were encoded on the light strand, others were encoded on the heavy strand.

Following Sangster and Luksenburg (2020), we verified the identity and integrity of our mitogenome sequence of *L. chinensis* with reference sequences of three commonly used markers in fish systematics: NADH dehydrogenase subunit 1 (ND1, 975 bp; 63 sequences of Salangidae, incl. three of *L. chinensis*), part of cytochrome oxidase subunit I (COI, 655 bp; 193 sequences of Salangidae, incl. five of *L. chinensis*), and cytochrome *b* (Cyt *b*, 1141 bp; 307 sequences of Salangidae, incl. three of *L. chinensis*).

In order to explore the evolutionary status of *L. chinensis* within the Salangidae, a Neighbor-Joining phylogenetic tree was constructed by MEGA7 (Kumar et al. 2016) based on the complete mitochondrial genome sequences of 10 species of Salangidae and two species (*Plecoglossus altivelis*, *Osmerus mordax*) as the outgroup (Figure 1). Except for *L. chinensis*, mitochondrial genome sequences of other species were downloaded from the NCBI GenBank. The phylogenetic tree showed that the 10 species of Salangidae clustered into three major clades: *Protosalanx hyalocranius*, *Protosalanx chinensis*, *Neosalanx anderssoni*, *Neosalanx tangkahkeii*, and *Neosalanx taihuensis* in clade1, *Leucosoma chinensis*, *Salanx ariakensis*, *Salanx cuvieri,* and *Hemisalanx brachyrostralis* in clade2, and *Salangichthys microdon* in clade3. The phylogenetic relationships inferred in our study based on mitogenome sequences were very similar to those obtained in a previous study based on cytochrome *b* sequences (Zhang et al. 2007).

**Figure 1.**
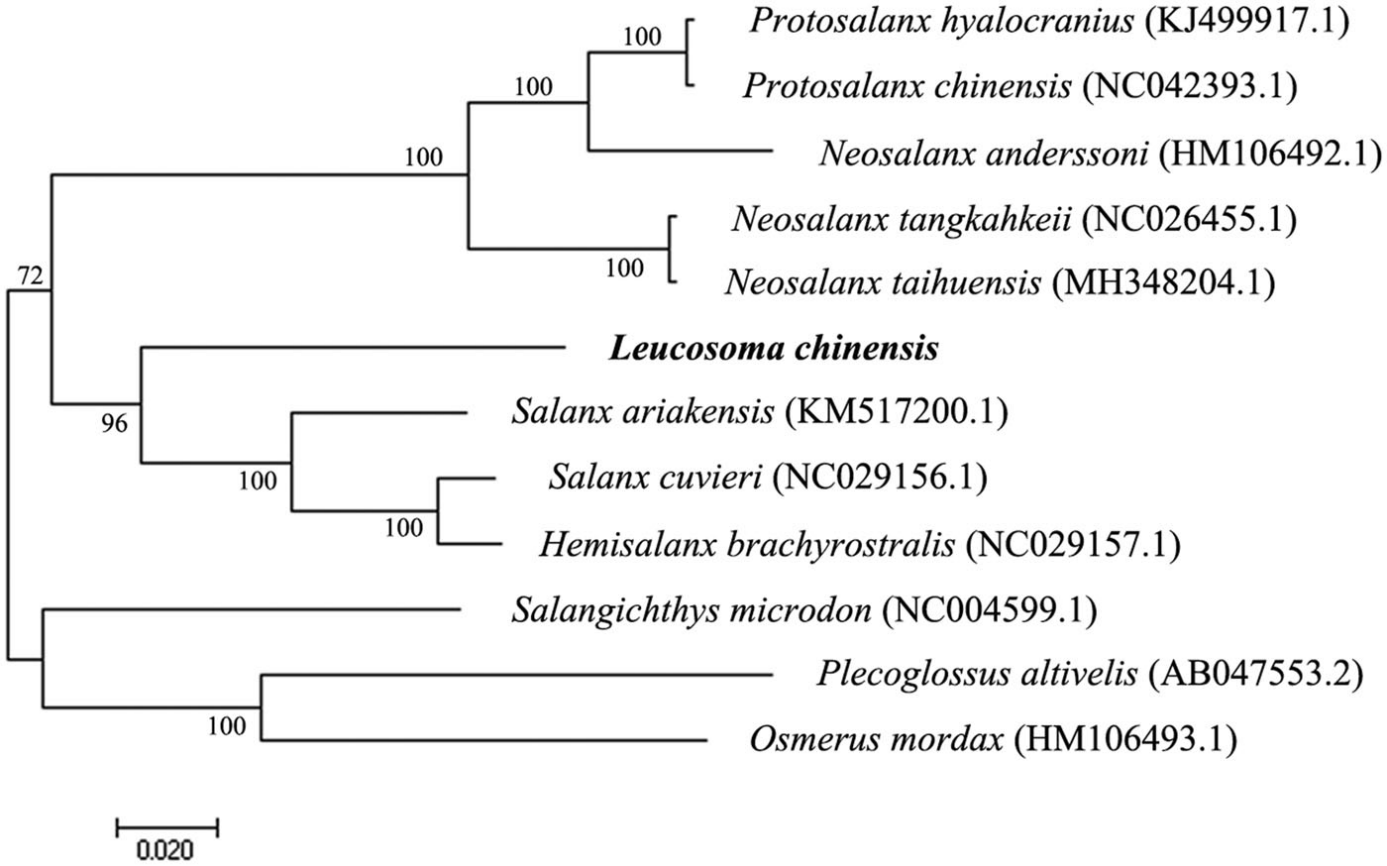
Neighbor-Joining phylogenetic tree of mitochondrial genome sequences of ten species of Salangidae and two outgroups (*Plecoglossus altivelis*, *Osmerus mordax*). The *Leucosoma chinensis* sequence obtained in the present study is shown in bold.

## Data Availability

The data that support the findings of this study are openly available in GenBank of NCBI at https://www.ncbi.nlm.nih.gov, reference number MW131880.
